# Mutagenic Consequences of Sublethal Cell Death Signaling

**DOI:** 10.3390/ijms22116144

**Published:** 2021-06-07

**Authors:** Christine J. Hawkins, Mark A. Miles

**Affiliations:** 1 Department of Biochemistry and Genetics, La Trobe Institute for Molecular Science, La Trobe University, Bundoora, VIC 3086, Australia; c.hawkins@latrobe.edu.au; 2 School of Health and Biomedical Sciences, RMIT University, Bundoora, VIC 3083, Australia

**Keywords:** DNA damage, DNA repair, mutagenesis, apoptosis, necroptosis, ferroptosis, pyroptosis, second malignant neoplasms, therapy-induced cancer

## Abstract

Many human cancers exhibit defects in key DNA damage response elements that can render tumors insensitive to the cell death-promoting properties of DNA-damaging therapies. Using agents that directly induce apoptosis by targeting apoptotic components, rather than relying on DNA damage to indirectly stimulate apoptosis of cancer cells, may overcome classical blocks exploited by cancer cells to evade apoptotic cell death. However, there is increasing evidence that cells surviving sublethal exposure to classical apoptotic signaling may recover with newly acquired genomic changes which may have oncogenic potential, and so could theoretically spur the development of subsequent cancers in cured patients. Encouragingly, cells surviving sublethal necroptotic signaling did not acquire mutations, suggesting that necroptosis-inducing anti-cancer drugs may be less likely to trigger therapy-related cancers. We are yet to develop effective direct inducers of other cell death pathways, and as such, data regarding the consequences of cells surviving sublethal stimulation of those pathways are still emerging. This review details the currently known mutagenic consequences of cells surviving different cell death signaling pathways, with implications for potential oncogenic transformation. Understanding the mechanisms of mutagenesis associated (or not) with various cell death pathways will guide us in the development of future therapeutics to minimize therapy-related side effects associated with DNA damage.

## 1. Introduction

Over the years, much research has been invested into understanding and defining the molecular mechanisms of various cell death pathways. A better understanding will enable us to develop effective therapeutics to combat diseases that are underscored by dysregulated cell death. Cell death pathways ensure tissue homeostasis and limit the pathology caused by various internal and external stresses (such as infection or radiation). A variety of mechanisms exist by which a cell can regulate its demise. Redundant cellular self-destruction pathways may act as failsafe switches for cells in contexts where blocks prevent sufficient propagation of one or more cell death pathways. 

Our knowledge of apoptotic cell death is extensive. Apoptosis is known for its “silent” mode of cell destruction without triggering immune activation. It is crucial during embryonic development, for maintaining tissue homeostasis under threatening conditions, and for the removal of cells that have reached their lifespan [1]. Perturbations in cells’ responsiveness to cell death signaling can lead to disease states, many of which are driven by mutations that fuel this dysregulation. Mutations arise upon incorrect repair of damaged DNA. Some mutations are lethal to the cell: the loss of function of an essential protein or widespread genomic alterations, while other (possibly subtle) mutations can be tolerated and passed on by living cells through subsequent cell divisions. The genomic instability brought on by various mutations may prime cells to a “mutation-prone” state, further allowing the acquisition of more mutations and facilitating oncogenic transformation. Cells tend to activate cell death responses upon detection of DNA damage when the damage is too extensive to be appropriately repaired by DNA repair pathways. This mechanism is exploited by various genotoxic anti-cancer therapies. In this aspect, cell death pathways act to protect tissues by limiting the transference of potentially oncogenic mutations to daughter clones. However, there is increasing evidence demonstrating that activation of sublethal cell death signaling pathways, in particular apoptotic signaling, in the absence of direct DNA-damaging stimuli, can promote genomic instability in cells that fail to die. In this review, we will discuss the mutagenic consequences associated with incorrect DNA repair and “failed” apoptosis, with particular emphasis on oncogenesis. We will look at DNA damage from a different viewpoint: extensive DNA damage initiated by radiation or chemotherapeutics initiates cell death, but nucleases activated during apoptosis (or some other forms of cell death) can also inflict genomic damage that may be mis-repaired. Hence, mutagenesis can occur in a cell surviving cell death signaling. The characterization of other regulated forms of cell death such as necroptosis, pyroptosis and ferroptosis and their implication in pathological disease is still ongoing. Data illustrating the ability of cells to withstand sublethal activation of these other pathways is emerging. We will therefore discuss the current evidence that addresses potential mutagenic consequences resulting from apoptosis and other cell death pathways. Further understanding the mutagenic capacities of cell death signaling pathways will help guide us in developing therapeutic strategies against diseases so that the risks of mutagenesis are minimized.

## 2. Mutagenic Consequences of DNA Mis-Repair

### 2.1. Repair of DNA Double-Strand Breaks

Errors in replication, exposure to genotoxic agents (e.g., ionizing radiation or certain chemotherapy drugs), and biological processes such as meiosis and V(D)J recombination create DNA double-strand breaks (DSBs). DSBs are lethal DNA lesions, as extensive damage signals the activation of key DNA damage response (DDR) elements to promote their repair or, if irreparable, senescence or cell death. This fatal consequence of widespread DSBs is exploited by traditional chemotherapies and radiotherapy to eliminate cancerous cells. Detection of DSBs begins with the association of the Mre11-Rad50-Nbs1 (MRN) complex at sites of damage [2]. This activates stress kinases—primarily ataxia telangiectasia mutated (ATM), ATM and Rad3-related kinase (ATR), and DNA-dependent protein kinase (DNA-PK)—which phosphorylate over 700 target proteins to direct the response to damage and catalyze repair [3]. ATM activity is often critical for the initiation of downstream DDR signaling pathways. Defects in MRN components hinder ATM signaling and prevent sufficient DNA repair, highlighting the importance of this kinase in responding to DNA damage [4]. Activated ATM is recruited to DSBs, probably via direct interaction with the Nbs1 C-terminal domain [5], where an autophosphorylation event at serine 1981 retains active ATM monomers at the breakage site [6]. Subsequent phosphorylation of histone 2AX at serine 139 (γH2AX) by ATM and/or other kinases is a key event that facilitates the assembly of DDR components and repair, and is often exploited experimentally as a marker for the presence of DSBs and DDR signaling [7]. 

An important function of this initial activation of DNA stress sensors is to regulate the cell cycle, to prevent the transmission of damaged DNA to daughter cells during mitosis [8]. Key phosphorylation ATM targets, Chk2 (primarily at threonine 68) and p53 (at serine 15), stop cells with damaged DNA from entering S-phase by activating the G1/S cell cycle checkpoint. Chk2 enables phosphorylation of Cdc25A phosphatase, which normally activates cyclin-dependent kinase 2 (Cdk2), needed for DNA synthesis, and targets it for proteasomal degradation to prevent the cell’s progression through S-phase [9]. Chk2 also assists ATM-mediated p53 activation by phosphorylating p53 to upregulate p21^Waf1/Cip1^ and sustain G1/S arrest [10,11]. Stretches of single-stranded DNA (ssDNA), such as those that occur at stalled replication forks, are bound by replication protein A (RPA) and subsequently recruit ATR and ATR-interacting protein (ATRIP) complexes [12]. ATR signaling pathways are often mediated through Chk1 and promote DNA stabilization, the restart of stalled replication forks, and cell cycle arrest. The G2/M checkpoint is primarily under the control of ATR and (to a lesser extent) ATM under these conditions to prevent premature entry of cells into mitosis and avoid cells dividing with damaged DNA [13]. This involves Chk1-mediated phosphorylation of Cdc25C phosphatase and subsequent binding to 14-3-3 proteins to inhibit Cdc2-mediated entry into mitosis [14]. Cdc2 can also be transcriptionally regulated by p53 to control entry into mitosis [15]. Mitotic death, driven by mitotic catastrophe, describes the death of a cell during incomplete mitosis [16]. Cells that evade mitotic catastrophe can exit mitosis without metaphase–anaphase transition (mitotic slippage), potentially harboring tetraploid genomic content [17]. Progression through mitosis after improper chromosomal segregation during anaphase can generate cells with aneuploid genomes [18,19]. Due to this, ineffective responses to DNA damage due to ATM, ATR, p53, or checkpoint kinase dysfunction can affect correct progression through the cell cycle, and thus may lead to oncogenic consequences. For instance, tumor cells with abnormal genomic content can exhibit perturbed gene expression, which facilitates intra-tumoral heterogeneity and may select for subclones with increased malignancy or resistance to therapy [20]. 

The direct repair of DSBs in mammals mainly occurs via two evolutionary conserved pathways. Expression levels of key components and/or the cell cycle phase in which DSBs are present dictate the repair pathway that a cell may rely on [21]. Non-homologous end joining (NHEJ) repair often occurs during interphase, is fast and efficient, and involves re-ligation of broken DNA ends, irrespective of sequence homology, hence it is prone to error [22]: NHEJ repair can give rise to small- and large-scale deletions as well as gross chromosomal rearrangements such as translocations [23]. ‘Classical’ NHEJ (C-NHEJ) repair begins with the binding of the Ku70/Ku80 heterodimer to broken DNA ends, which recruit and activate the DNA-PK catalytic subunit (DNA-PKcs), forming a complex known as the DNA-PK holoenzyme [24,25]. DNA-PKcs can phosphorylate many target proteins, including H2AX. Its role in NHEJ is to associate with the nuclease Artemis, which then carries out endonucleolytic cleavage of 5′ and 3′ overhangs. Resealing of DNA ends is then under the control of the XRCC4-DNA ligase IV complex [26]. Active XRCC4-DNA ligase IV can also associate with the DNA-PK holoenzyme to further stimulate Artemis activity as a positive feedback for NHEJ repair [27]. In contrast to C-NHEJ, which involves minimal processing of the DSB termini prior to ligation, ‘alternative’ NHEJ can proceed in the absence of sufficient C-NHEJ activity. Ultimately, this version of NHEJ involves resection of up to 100 nucleotides at the breakage ends until regions of microhomology (typically 1–3 nucleotides) are revealed [28]. Microhomology has been implicated in V(D)J recombination events, leading to genetic diversity [29]. 

In contrast to error-prone NHEJ, homologous recombination (HR) repair is highly accurate as it relies on an intact homologous donor sequence [30]. HR readily repairs DSBs in S and G2 phases because sister chromatids are easily accessible. Matching sequences on homologous (or even non-homologous) chromosomes can also be used as templates for HR—indeed, this process is crucial for chromosomal assortment during meiosis—but sister chromatids are favored (when present) for HR within somatic cells due to their proximity [31]. HR is achieved by initially resecting broken DNA ends, giving rise to ssDNA with exposed 3′ overhangs or when replication forks stall. These ssDNA are coated by RPA proteins, which attract the ATR kinase and recombination mediators such as RAD52, the RAD55-RAD57 heterodimer, and BRCA2 [32,33,34]. Recruitment of these mediators helps to displace RPA from the DNA strand and allows RAD51 binding. Once bound, RAD51 polymerizes in the presence of ATP with the help of BRCA2 to form helical nucleoprotein filaments, which enable homology search and DNA joint formation [35]. RAD51 also functions in replication fork reversal [36]. DNA helicases promote strand displacement and synthesis-dependent strand annealing, while endonucleases resolve the Holliday junctions formed on the second DSB end. The assembly and preservation of the RAD51 nucleoprotein filament is guided by protein complexes that consist of RAD51 paralogs and BRCA2. Accordingly, defects in any of these proteins can reduce the efficiency of accurate repair [37]. Due to the requirement for a homologous template, mitotic and meiotic events rely on HR for appropriate crossover and preservation of genetic information free of mutations.

### 2.2. Oncogenic Consequences of DNA Mis-Repair 

The mis-repair of damaged DNA or cell entry into mitosis with unrepaired damage can give rise to genomic instability through the loss or gain of genomic content, or as a consequence of chromosomal rearrangements. This could increase the chance of malignancy [38]. Germline mutations in key genes involved in responding to or repairing DNA damage can result in a number of heritable disorders that are often associated with an increased risk of malignancy [39,40]. The penetrance of inherited conditions associated with particular gene variants may vary and certain familial syndromes may require more than one polymorphic variant to observe a phenotypic effect. For example, the occurrence of sarcomas has been associated with over 20 different inherited syndromes [41]. Mutations in unrelated genes may also give rise to similar risk phenotypes as they may serve as crucial components to the function of highly conserved pathways that help maintain genomic stability. Breast cancers, for example, arise frequently in individuals bearing germline mutations in *BRCA1* or *TP53* [42], which are phosphorylation targets of ATM, which itself when mutated also confers a risk for breast cancer [43]. Despite the predisposition of familial cancer syndromes to different cancers, the predicted allelic frequency of these pathogenic variants is low within the general population, suggesting the involvement of other factors contributing to cancer development [44]. As discussed below, therapeutic exposure to DNA-damaging agents may pose carcinogenic risks to a cell that does not succumb to death, perhaps especially in cells with unstable genomes because of impairments in the ability to accurately repair DNA. In this case, DNA-damaging therapies may enhance tumorigenesis, as non-cancerous cells harboring DNA repair defects would be less likely to fix this damage, therefore prompting incorrect repair and facilitating mutagenesis [45].

More than a quarter of cancer diagnoses are now made in survivors of previous cancers [46]. Childhood cancer survivors can be up to six times more likely to succumb to a subsequent cancer, and this risk is also rising in adult survivors [47,48,49,50,51]. These subsequent malignancies emerge independently from a patient’s initial cancer, are often more aggressive, less responsive to treatment and present with poorer prognosis [52,53,54,55]. Genotoxic anti-cancer treatments have been implicated in tumorigenesis, and this represents a concerning late effect known as ‘therapy-related’ cancers, although treatment-independent factors also contribute [56]. As such, therapeutic exposure is considered a risk factor for cancer development [55]. Survivors of testicular cancer [57] or osteosarcoma [58] were more likely to develop subsequent cancers if treated with chemotherapy than surgery. Likewise, the incidence of secondary breast cancers was higher in patients who received chest radiation or (to a lesser extent) alkylator and/or anthracycline chemotherapy compared to patients who had not received these treatments [51,59]. The degree of exposure to these therapies appears to also influence the risk of acquiring some second cancers [60]. For instance, the incidence of second malignancies was at least 4 times greater in high-risk neuroblastoma patients who were administered chemotherapeutic regimens compared to low-risk patients who received minimal chemotherapy exposure [61].

A mutational signature generated by different genotoxic treatments reflect the mechanisms by which these treatments damage DNA and the repair processes that correctly (or incorrectly) repair the damage [62]. Additionally, such treatments can have direct effects on DNA by chemically modifying the structure of nucleobases (such as alkylation and oxidation), leading to incorrect pairing of complementary bases and the alteration of genomic sequence [63]. Chromosomal abnormalities characterizing therapy-related acute myeloid leukemia or myelodysplastic syndrome (t-AML/MDS) have been extensively associated with agents that alkylate DNA or target topoisomerase-II proteins [64]. Sarcoma patients receiving high-dose doxorubicin in combination with the alkylators ifosfamide and cyclophosphamide were reportedly 16 times more likely to develop t-AML/MDS than patients who received low-dose doxorubicin without alkylator treatment [65]. Similarly, t-AML/MDS was more frequent in survivors of neuroblastoma treated with both epipodophyllotoxins and alkylating agents [66]. Genes encoding crucial hematopoietic growth factors located on chromosomes 5 and 7 are commonly deleted following the mis-repair of alkylated DNA, increasing the risk of leukemic development [67]. Balanced translocations involving the mixed lineage leukemia locus (*MLL*) at 11q23 are highly prevalent in these t-AML/MDS cases [68]. The frequency of characteristic translocations generating the fusion genes *MLL-AF9* t(9;11), *MLL-AF4* t(4;11), *PML-RARA* t(15;17), *AML-ETO* t(8;21) and *MYH11-CBFB* inv(16), are higher in patients treated with topoisomerase-II poisons, often reported for etoposide, compared to patients treated with other therapies [69,70,71]. This is probably because the cleavage region of translocated sequences commonly falls within a breakpoint cluster region (bcr) that encompasses known nuclear matrix attachment regions, DNase hypersensitive sites, and topoisomerase-IIα cleavage sites [72]. The location and size of the bcr in de novo cancers appear to localize towards the centromeric end of the chromosome, but therapy-related cases tended to concentrate towards the telomeric 1kb region of this bcr, which are closer to topoisomerase-II cleavage sites, implicating these oncogenic DNA lesions as a direct consequence of topoisomerase action [73]. In support of this, analysis of acute promyelocytic leukemia (APL) that developed after treatment with mitoxantrone occurred within a tight 8bp cluster ‘hotspot’ within the *PML* intron 6, a region that topoisomerase-II proteins could cleave upon treatment with etoposide or doxorubicin [74,75]. These *PML* breakpoints were not detected in de novo APL while clustering of *RARA* breakpoints occurred close to a topoisomerase-II consensus sequence and was twice as prevalent in t-APL than de novo APL [75]. Next-generation sequencing has recently been used to profile gene mutations in patient samples to draw comparisons between de novo and therapy-related AML/MDS cases, highlighting a difference in the mutation profile between them and supporting the mutagenic potential of these drugs [76,77,78]. 

## 3. Mutagenic Consequences of Apoptotic Signaling

### 3.1. Caspases and Apoptosis

Apoptotic caspases consist of initiator (caspases-2, -8, -9, and -10) and executioner (caspases-3, -6, and -7) subclasses and are distinguished by the presence or absence of a pro-domain [79]. Initiator caspases contain pro-domains, such as the caspase-recruitment domain (CARD) or death effector domain (DED), which recruit the caspase to oligomeric activation platforms such as the apoptosome and death-inducing signaling complex (DISC), where it dimerizes and activates via induced proximity [80]. Active initiator caspases are able to cleave specific protein substrates, which include the linker regions between the small and large subunits of inactive dimeric executioner procaspases to stabilize their active sites [81]. Executioner caspases-3 and -7 can cleave an array of substrates that include but are not limited to: PARP (to limit DNA repair), ICAD/DFF45 (to activate the nuclease CAD/DFF40 which cleaves DNA into fragments), actin and gelsolin (to facilitate cytoskeletal reorganization), and nuclear lamins (to induce nuclear condensation) [82]. Caspase-3 appears to show the most potency in substrate cleavage during the execution phase of apoptosis [83,84]. Additionally, executioner caspases can also feed back to further enhance caspase activation [85]. Infection or exposure to pathogenic toxins can stimulate a caspase-mediated, non-apoptotic, inflammatory form of cell death known as pyroptosis (discussed in more detail later). Inflammatory caspases-1, -4 and -5 ultimately promote loss of membrane integrity to create an osmotic influx, resulting in cell lysis, release of cellular contents, and activation of an immune response [86,87]. A secondary pyroptotic process can also be initiated by apoptotic executioner caspases in apoptotic cells that fail to disassemble and be cleared [88].

Intrinsic (mitochondrial) apoptotic signaling is triggered by DNA damage and other internal cellular stresses such as viral infection, growth factor withdrawal, and hypoxia [89]. The tumor suppressor p53 often modulates this response: it is normally kept at low levels in healthy cells through proteasomal degradation by the E3 ubiquitin ligase MDM2, but stress signals activate post-translational modifications to stabilize p53 [90]. Accumulation of p53 proteins in the cell suppresses cellular growth by transcriptional upregulation of genes essential for cell cycle arrest, senescence, or apoptosis [91]. For instance, DNA damage causes p53-mediated induction of p21^Waf1/Cip1^ expression to bind to cyclin-cdk complexes and inhibit phosphorylation of the retinoblastoma protein (Rb) to arrest cells in G1 and allow for DNA repair [92]. If the DNA damage is too extensive for repair, p53 induces transcription of pro-apoptotic proteins such as p53-upregulated modulator of apoptosis (PUMA) [93] and Bax [94]. These proteins, along with other pro-apoptotic Bcl-2 relatives such as Bim, Bid, Bad, and Noxa, alleviate the pro-survival properties of Bcl-2, Bcl-_xL_, and Mcl-1 to promote Bax/Bak-dependent mitochondrial outer membrane permeabilization (MOMP). Subsequent release of cytochrome *c* from the mitochondria and its association with cytosolic apoptotic protease activating factor 1 (Apaf-1) provides a caspase-9 activating platform via the apoptosome [95]. Caspase-9 can then cleave procaspase-3 to execute apoptosis. Another mitochondrial apoptogenic factor is also released from the mitochondria: second mitochondria-derived activator of caspases/direct IAP binding protein with low pI (Smac/DIABLO), which neutralizes the caspase-inhibiting activity of inhibitor of apoptosis proteins (IAPs) to also promote cell death [96]. X-linked IAP (XIAP) can prevent caspase-3 or -7 activation by either competitive or non-competitive binding to the caspase active site [97], while it can also sequester caspase-9 monomers to limit its overall catalytic activity [98]. Smac/DIABLO also has affinity for cellular IAPs 1 and 2 (cIAP1/2), promoting their proteasomal degradation and activating non-canonical NFκB signaling and/or formation of death signaling complexes downstream of the tumor necrosis factor receptor 1 (TNFR1) [99].

Activation of the extrinsic pathway occurs upon the binding of external ligands to cell surface death receptors belonging to the tumor necrosis factor (TNF) family of receptors [100]. Interaction of TNF α, Fas ligand (FasL/CD95L), or tumor necrosis factor-related apoptosis inducing ligand (TRAIL/Apo2L) to their corresponding death receptor leads to the formation of a cytosolic DISC, which acts as a caspase-8/-10 activating platform. Interaction between the death domains of the receptor and the adaptor molecule FADD promotes the recruitment of procaspase-8 (or -10) or cellular FLICE inhibitory protein (FLIP), an inhibitor of caspase-8 and -10, at the DEDs [101,102]. Executioner caspases can be directly activated by caspase-8 in type I cells, such as lymphocytes, whereas type II cells, such as hepatocytes, require caspase-8-mediated cleavage of the BH3-only protein Bid to tBid to propagate apoptotic signaling via MOMP and the apoptosome [103]. The levels of XIAP within the cell appear to dictate the signaling pathways occurring in such cell types [104]. 

### 3.2. Functions of Sublethal Apoptotic Signaling

Early studies using single-cell fluorescence resonance energy transfer (FRET) assays implied that when a cell commits to apoptosis, as indicated by caspase activation and/or mitochondrial damage, it occurs as an “all or nothing” event [105]. Cells with compromised mitochondria, irrespective of active caspases, fail to divide clonogenically, highlighting the critical role for mitochondrial integrity in cell survival [106]. However, research over the last few decades has accumulated evidence that supports various non-apoptotic roles of caspases in promoting cell differentiation and processing [107]. Transient activation of apoptotic caspases is essential for encouraging the maturation and differentiation of various hematopoietic progenitor cells [108,109,110]. For instance, active caspase-3 was detected in proliferating T cells following antigen presentation [111,112], while caspase-8 promoted macrophage differentiation by cleaving RIPK1 and limiting NFκB activation [113]. Genetic ablation of caspase-3 or -9 in vivo resulted in a lower proportion of mature myeloid and lymphoid cells and more undifferentiated hematopoietic stem cells compared to mice that were caspase-proficient [114,115]. Mouse embryonic stem cells (mESCs) lacking caspase-3 also displayed defective differentiation [116]. Furthermore, caspase-3 was reportedly essential for complete myogenic differentiation in vitro [117,118]. Caspase-3-deficient myoblasts lacked myotube formation and downregulated expression of skeletal muscle-specific differentiation markers [118], while complete incapacitation of caspase-3 affected skeletal myoblast differentiation [119]. Additionally, caspase-3 cleavage of ICAD and subsequent nuclease activation of CAD was observed in terminally differentiating skeletal muscle [117], allowing for enhanced gene expression and DNA repair to facilitate cell survival [120].

Caspases have also been implicated in the crafting and processing of specialized cell types. Caspase-3, -6, and -9 are involved in axonal or dendritic pruning upon nerve injury or nerve growth factor (NGF) withdrawal, and XIAP-mediated regulation of caspase-3 correlated with the rate of degradation [121,122,123]. Axons from caspase-3 or -6-deficient mice were maintained upon withdrawal of NGF, which would otherwise promote degradation [121,124]. Interestingly, even though cellular degradation was localized to the protruding/degenerating axons alongside active caspases [125,126], upstream signaling and caspase-activating machinery were detected within the neuronal cell bodies, and were regulated by PUMA and Foxo3a/c-Jun transcription along with the loss of pro-survival Akt signaling [125]. This suggests that caspases are either activated at the degenerating axonal site or are activated at but not retained in viable cell bodies. It is not yet known how the caspase-cascade can dictate the destruction between the neuronal compartments. The establishment of lens transparency within the eye also involves coordinated degradation of eye lens fiber components mediated by apoptotic regulators [127]. Activated Bax, cytochrome *c* release, and apoptosome formation were observed in regions of lens differentiation or during de-nucleation, all the while plasma membranes remained intact [128,129]. This process could be mediated upstream by heat shock transcription factor 4 (HSF4) as HSF4-deficient zebrafish presented with cataracts and showed lower p53 and activate caspase-3 levels [130]. Caspase-3 and -6 were also catalytically active in mouse and rat lenses extracted during periods of organelle loss [131], and low-level caspase activity was detected in differentiating lens fibers from chick embryos [129]. Interestingly, Zandy et al. [132] failed to observe any difference in the architecture of differentiated lenses in single knockout caspase-3, -6, or -7 mice or caspase-3/-6 double knockout mice, and only mice deficient for caspase-3 presented with cataracts.

### 3.3. Molecular Mechanisms of Sublethal Caspase Activation

Given the physiological evidence for various non-apoptotic roles of otherwise classified “apoptotic” caspases, what mechanisms exist that allow a cell to survive apoptotic signaling given that caspase activation was originally considered the “point of no return” in a cell’s fate? In order for executioner caspases to execute cell death, cleavage of vital proteins that are essential for cell survival, such as cell scaffolding proteins, signal transduction, and transcription-regulatory proteins, and proteins involved in DNA repair, must occur at a rate that exceeds the cell’s ability to replenish them. Caspase cleavage commonly leads to the loss-of-function of target substrates. For example, caspase cleavage of PARP renders the protein non-functional. Thus, a reduction in intact (functional) PARP would slow down the rate of DNA repair in favor of apoptosis, while the presence of more PARP molecules would accelerate the rate of survival [133]. Therefore, boosting protein synthesis could ensure that sufficient levels of proteins essential for survival exist and, if maintained, could allow the cell to persist despite concurrent caspase-mediated cleavage of a small proportion of those proteins. Conversely, some caspase cleavage events are gain-of-function, for instance the cleavage of Bid to tBid (to activate its pro-apoptotic ability to induce Bax/Bak-mediated MOMP). Bcl-2 overexpression can prevent tBid-mediated death, but not conversion of Bid into tBid [134], indicating that Bcl-2 overexpressed cells may withstand some level of caspase-cleaved tBid. The activation of caspase-3 is a rapid process that occurs almost immediately after MOMP. Cells over expressing Bcl-2 or XIAP display a slower caspase-3 activation rate and minimal substrate cleavage, indicating that XIAP can restrict caspase activity to sublethal levels in cells [135,136]. To avoid the persistence of sublethal levels of active caspase-3, a positive feedback loop driven by caspase-3 can occur to activate caspase-9 and the apoptosome, thereby further permitting the release of Smac from permeabilized mitochondria to relieve XIAP inhibition and augment caspase-3 activation [85]. Based on this, if MOMP-independent activation of caspase-3 occurred (such as upon extrinsic activation in type-I cells) in a slow manner, a delayed caspase-mediated MOMP and subsequent Smac release might ensue that could be sufficiently neutralized by XIAP to evade the extent of substrate cleavage required for death. Evidence for this has been published: TRAIL-induced caspase-8 activity persisted in Bid-depleted cells without causing immediate cell death, consistent with the lack of MOMP and executioner caspase activity [137], and illustrating that a cell could experience some degree of active caspase in the absence of MOMP and death. Reinforcing this notion, apoptotic signaling occurred slower in differentiating mESCs as opposed to a rapid onset in apoptotic mESCs [138].

A lethal apoptotic stimulus should kill all cells of a clonal origin as they all should be genetically identical; however, a subset of cells may survive, and this cell-to-cell variability in sensitivity defines a type of “fractional cell killing”. The heterogeneity in cell sensitivity has been reported to occur following death receptor activation and exposure to chemotherapeutic drugs, and may be the result of natural fluctuations in levels of pro- and anti-apoptotic proteins [139,140,141,142]. Cells surviving TRAIL exposure became transiently resistant to a second round of exposure, unlike staurosporine treatment which generated a mixed population of sensitive and resistant surviving cells [140]. Intriguingly, this resistance could be reversed as cells eventually restored the same degree of fractional killing and eventually reset to a state almost identical to a naïve cell. The time at which caspase-8 activity reaches a threshold within the cell appeared to determine whether the cell lived or died following TRAIL treatment: caspase-8 activity rose more slowly in cells that survived compared to cells that died rapidly [141]. HeLa cells (type-II cells) were reported to enter a state of “delay” following TRAIL exposure prior to MOMP and executioner caspase activation, illustrating a period during which initiator but not effector caspases are active [136]. In this situation, a small number of executioner caspase-specific substrates were processed prior to the cell reaching the caspase-8 activation threshold that was required for the rapid substrate cleavage, implying that substrate cleavage can still occur, albeit less efficiently, even when cells have not committed to apoptosis [136]. The caspase-8 activation threshold changed when Bcl-2 and Bcl-_xL_ levels were reduced, consistent with mitochondrial ‘priming’ and MOMP further driving caspase activation [137,141]. Another level of control could be achieved via proteasome-mediated degradation of caspases to also limit their activation. Inhibition of the proteasome by bortezomib or MG-132 maintained active caspase levels and enhanced substrate cleavage upon pro-apoptotic stimuli [136,141]. An association between the proteasome and caspase-3 degradation has been reported in the presence of XIAP: caspase-3 was ubiquitinated when bound to XIAP, which targeted it for proteasomal degradation in unstimulated cells [143]. In this way, alterations in proteasome function may allow for a certain level of caspase-3 to overcome XIAP inhibition and promote some level of substrate cleavage. Despite these links, proteasome inhibition is likely to affect the degradation of multiple proteins, so it is difficult to conclusively define the mechanisms by which proteasome activity and inhibition influence apoptotic signaling and cell fate [144].

Fractional killing upon DNA damage appears to be largely determined by the rate of p53 accumulation above an apoptotic threshold, such that cells undergo apoptosis when the p53 threshold is achieved quickly, whereas cells survive when p53 activation is delayed [142,145]. The maximal levels of p53 that are eventually attained, however appear similar among apoptotic and surviving cells and both states activate pro-apoptotic and cell cycle arrest genes, further implying that it is the rate of p53 activation that may determine cell fate [142]. It was postulated that sustained activity of IAPs (chiefly XIAP and cIAP1/2) limited p53-dependent apoptosis, so changes in IAP levels following DNA damage may control the p53 pro-death threshold [142], much like the XIAP-mediated control of the caspase-3 threshold discussed above. 

Coined by Tang et al. [146] “anastasis” describes the reversal of apoptosis in which cells that display classical apoptotic hallmarks such as cell shrinkage, nuclear condensation, and mitochondrial fragmentation can retain viability and proliferative ability following removal of the stimulus. Anastasis has been described in both primary and cancerous cells and has been proposed to rescue cells from crisis [146,147]. In key experiments, intrinsic apoptosis was induced following incubation with ethanol, DMSO or staurosporin, or extrinsic apoptosis was triggered by exposure to TNFα and cyclohexamide [148]. Cells recovered after MOMP and caspase-3 activation, although they eventually succumbed to death if left in the presence of the inducer, most likely due to full execution of apoptosis mediated by caspase-3 cleavage of substrates. Sun et al. [148] reported that new RNA synthesis occurred immediately upon cell recovery, describing an initial upregulation of genes involved in pro-survival and cell cycle pathways followed by genes involved in post-translational activities such as RNA transport, ribosome biogenesis, focal adhesion, and regulation of actin cytoskeleton. Consistent with the sufficient cell recovery time in the absence of extensive substrate cleavage mentioned earlier, the transcription of pro-survival Bcl-2 and IAPs would restrict and further limit caspase activity, while new protein synthesis would be essential for boosting intact substrate levels and reversing the cellular changes resulting from their proteolysis and inactivation. The authors indicated that many of these pathways were also active during wound healing and hypothesized that cells utilize this recovery process to reduce permanent tissue damage after transient injury. 

Understanding the factors that permit cells to survive despite harboring active caspases requires sensitive tools for monitoring caspase activity. A number of biosensors are available to detect, monitor, and track real-time caspase activity at a single cell level in cultured cells or animal tissues [149]. These systems often utilize FRET-based analysis, for example SCAT to detect the FRET from ECFP to Venus fluorescence [150], or localization-based tags such as Apoliner [151]. It can be difficult, however, to identify whether the cells containing active caspases have survived and proliferated. More recently, the in vivo biosensor system CaspaseTracker allows for the permanent marking of cells that presently or previously exhibited caspase activity [152,153]. This system has been described in *Drosophila,* where a fluorescent signal denotes a history of caspase activation within that cell and detection up to 10 days after repression of the biosensor [152]. The use of such methods will be critical to fully understanding the extent of sublethal caspase signaling and its effects in vivo.

### 3.4. Caspase-Dependent DNA Damage Via Nucleases

**Figure 1:**
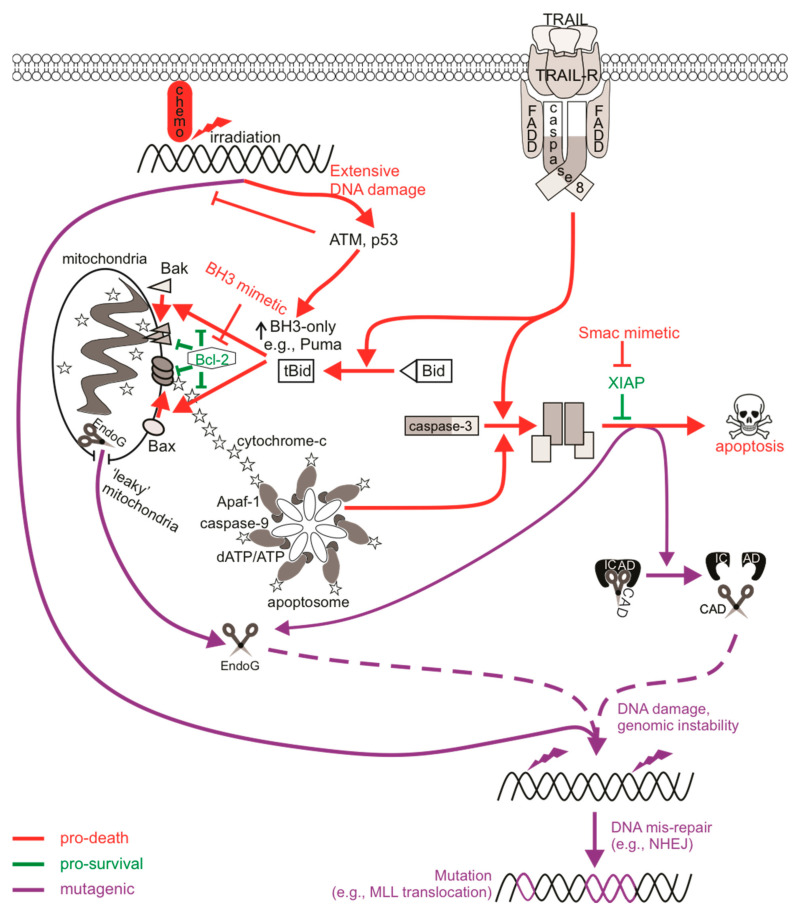
The mutagenic potential of apoptotic signaling. Chemotherapy drugs induce DNA damage that can be recognized by DNA damage sensors such as ATM to direct cell signaling towards DNA repair or death. An insufficient response (for instance, when the ATM function is defective) may encourage the activation of low-fidelity repair pathways such as non-homologous end joining (NHEJ) that are error-prone, increasing the likelihood of genomic mutations. Cell death may also be primed for, leading to p53-mediated upregulation of pro-apoptotic Bcl-2 proteins to induce Bax/Bak-mediated permeabilization of the mitochondrial outer membrane (MOMP), cytochrome *c* release, and the activation of caspase-9 via the apoptosome. Active caspase-9 can promote executioner caspase activation, which then cleave cellular substrates to induce apoptosis. Extrinsic activation of death receptors can also activate this caspase-cascade. Apoptosis-induced genomic fragmentation by caspase-activated DNase (CAD), which becomes active upon executioner-mediated cleavage of its inhibitor ICAD, can provoke mutations via NHEJ repair if sublethal levels of apoptosis are achieved. BH3 mimetics promote apoptotic cell death by relieving the inhibition of pro-survival Bcl-2 proteins. Some cells may experience “minority” MOMP, which describes the sublethal release of cytochrome *c* and caspase activation from “leaky” mitochondria. Mutagenesis upon “minority” MOMP can occur due to the release of endonuclease G (EndoG) from the mitochondria and its direct action on DNA or via caspase/CAD-dependent pathways. The mis-repair of nuclease-mediated DNA fragmentation could lead to oncogenic mutations such as chromosomal rearrangements that alter the *MLL* gene to increase the risk of acute myeloid leukemia.

Targeting specific points within apoptotic pathways may bypass classical blocks in apoptosis that are exploited by cancer cells, for instance mutant p53 or enhanced expression of pro-survival Bcl-2 proteins, which can drive cancer development and progression, and facilitate chemoresistance [154]. Molecules such as BH3-mimicking drugs or IAP antagonists directly engage apoptotic components to initiate cell death signaling, and as such (unlike chemotherapy or radiotherapy) do not need to cause DNA damage in order to elicit a cytotoxic response. This advantage could theoretically spare cells from the mutagenic mis-repair of damaged DNA inflicted by chemotherapy and radiotherapy, and hopefully avoid triggering therapy-related cancers in cured patients. Despite this initial hope, various reports have described DNA damage upon direct apoptotic signaling, illustrating that sublethal exposures can promote mutagenesis. Mechanistically, the genotoxic nature of sublethal apoptotic signaling has been attributed to apoptotic caspases, in particular executioner caspase-3, and apoptotic nucleases [155] (Figure 1). Executioner caspases proteolytically cleave a number of different substrates, but their cleavage of ICAD to release active CAD has direct effects on DNA integrity. ICAD normally sequesters CAD in an inhibitory complex in healthy cells, however CAD nuclease activity occurs upon caspase-mediated cleavage of ICAD [156]. CAD is responsible for late-stage cleavage of high molecular weight chromatin and promotes oligonucleosomal fragmentation and degradation of DNA, a characteristic of apoptosis in many cell types that facilitates clearance of apoptotic bodies and debris by phagocytes [157,158]. This process has been reported to occur in cancer cells in the absence of external apoptotic stimuli: constitutive sublethal caspase and nuclease activity within cancer cells promoted the continual generation of spontaneous DSBs, further driving tumorigenicity [159].

Activation of extrinsic apoptotic signaling via the ligation of TRAIL or Fas death receptors activated a DNA damage response [160] and provoked mutations in surviving cells [161]. Mutagenesis following exposure to TRAIL or proteasome inhibition was abolished in caspase-3/7- or CAD-deficient cells, or when caspases were chemically inhibited [161,162,163], indicating that the mutagenic signal propagated from direct activation of CAD by executioner caspases. Cells experiencing prolonged mitotic arrest also harbored detectable levels of DNA damage that was inhibited by caspase deficiency [164,165]. Indeed, CAD was implicated in the genotoxicity and mutagenicity of anti-mitotic drugs that induce a delay in mitosis [162,164,165]. Pro-survival Bcl-2 proteins normally prevent MOMP and cytochrome *c* release during mitosis to allow sufficient division and progression through the cell cycle. However, in response to microtubule poisons, which prolong the time that a cell remains in mitosis, Mcl-1 (and possibly other pro-survival relatives) is phosphorylated by CDK1-cyclin B and subsequently degraded, allowing for Bax/Bak-mediated cytochrome *c* release to activate caspases [166]. Microtubule poison-mediated apoptosis would occur once the cell exits mitosis, however it was reported that a partial apoptotic response occurred if the cell remained in a state of prolonged mitosis resulting in p53 induction and DNA damage [164]. In this model, Mcl-1 degradation facilitates the partial release of cytochrome *c* to activate caspases to sublethal levels which activate CAD and provoke DNA damage. The DNA damage activated ATM, DNA-PK and (to a lesser extent) ATR, which induced p53 stabilization and activation [167]. The caspase-mediated DNA damage during delayed mitosis therefore acted as a feedback stimulus to enhance the apoptotic response. Interestingly, vincristine-treated cells acquired DNA damage and harbored mutations via CAD mutagenesis [162], implying this re-enforcement of apoptotic signaling during prolonged mitotic arrest can be tolerated in some cells. 

In line with the concept of partial cytochrome *c* release from the mitochondria in otherwise viable cells, Ichim et al. [168] reported CAD-mediated genotoxicity in BH3 mimetic-treated cells with “leaky” mitochondria, resulting in sublethal MOMP (“minority” MOMP) and low-level caspase activity. Unlike CAD-mediated mutagenesis following death receptor ligation or during periods of prolonged mitotic arrest, whereby inhibition of DNA-PK (or ATM) activity severely reduced H2AX phosphorylation [160,162,165], DNA damage following antagonism of Bcl-2/Bcl-_xL_ by ABT-737 appeared to be dependent on expression of JNK1/2 [168]. JNK-dependent sublethal MOMP and subsequent caspase-mediated nuclease genotoxicity was also reported in naïve cancer cells [159]. This may suggest a difference in key DDR protein activation of certain DNA repair pathways depending on either cell type or strength of the stimulus. For instance, Ichim et al. [168] described transforming capabilities for ABT-737 under oncogenic cellular conditions, while Shekhar et al. [169] failed to detect mutations in clonogenically viable cells after sublethal treatment with the same drug, at least at concentrations that elicited Bax/Bak-dependent death. Exposure to ABT-263/Navitoclax (an orally available derivative of ABT-737) also failed to provoke DNA damage or mutations at clinically relevant concentrations, even when increases in caspase activity and apoptosis were detected [170,171]. It is possible for some but not all cell types to tolerate minority MOMP, given that loss of mitochondrial function often impacts a cell’s clonogenic competency. This may be due to varied expression of pro-survival proteins such as IAPs or Bcl-2 family members between the different cell types used in these studies, which may influence whether or not cells tolerate and recover from mitochondrial damage [172]. Minority MOMP and associated caspase/CAD-dependent mutagenesis was also implicated upon expression of the BH3-only protein BIK [173]. BIK-expressed or staurosporine-treated cells with low and high levels of caspase activity were sorted and clonogenic potential determined. Colonies failed to form following staurosporine exposure regardless of caspase levels, whereas at least 50% of cells maintained clonogenicity after BIK expression and caspase activation, implying partial MOMP within viable cells [173].

Endonuclease G (EndoG) is another nuclease that can promote DNA fragmentation, which can operate in the presence or absence of active caspases [174]. EndoG is localized within the mitochondria in healthy cells but can translocate to the nucleus upon MOMP to cleave single- or double-stranded DNA substrates [174]. There is evidence that sublethal executioner caspase activity can cause DNA damage via EndoG. MCF10A cells transduced with a caspase-3 reporter were live-cell sorted based on the magnitude of caspase activity following sublethal radiation exposure, and were found to maintain clonogenic potential despite containing active caspases. DNA damage and chromosomal aberrations were detected in these cells when caspase-3 or EndoG were present and functional [175]. Caspase-3 and EndoG were also implicated in mutagenesis driven by c-Myc over-expression [176]. Here, the frequency of γH2AX staining or chromosomal aberrations were enhanced when c-Myc was over-expressed, but only in caspase-3 proficient cells. In vivo c-Myc-induced tumorigenicity also required caspase-3 and EndoG, although a version of EndoG where the mitochondrial localization signal was substituted for a nuclear localization signal was enough to promote c-Myc tumorigenesis in caspase-3 knock-out cells, indicating that the nuclease function of EndoG acted downstream of caspase-3. Given that the reports implicating EndoG-induced genomic instability are accompanied by caspase activation, CAD would presumably also act and contribute to the DNA damage in these cells given the high preference of ICAD cleavage by caspase-3. Using CRISPR/Cas9, Liu et al. [159] assessed the contribution of both CAD and EndoG in caspase-mediated genotoxicity in cancer cells and found that deficiency of either or both nucleases reduced DNA damage, suggesting that chromatin fragmentation achieved by either nuclease was sufficient to activate a DNA damage response. Unlike CAD, EndoG can be active without the need for caspases (as MOMP can occur independently of standard apoptotic stimuli), and as such, EndoG-mediated DNA damage has been reported following serum starvation or caspase-independent radiation, leading to the induction of autophagy [177,178]. 

### 3.5. Oncogenic Consequences of Caspase Signaling

The pathological impact of apoptosis can have various effects on tumorigenesis and cancer. In the first instance, caspase activity within cells that do succumb to apoptotic death can offer oncogenic advantages. Dying cells were reported to release mitogenic signals, via active executioner caspases, which promoted the proliferation of surviving, neighboring tumor cells. This phenomenon, termed “apoptosis-induced proliferation”, has implications in tumor repopulation, post-therapeutic relapse, and oncogenic cellular evolution [179,180,181]. Many of the mechanistic studies of apoptosis-induced proliferation utilized *Drosophila* [182], in which exposure to cytotoxic therapies triggered caspase-mediated substrate cleavage, leading to the activation of JNK-dependent signaling and subsequent secretion of mitogens such as Wnt molecules or proliferative cytokines such as prostaglandin E_2_ (PGE_2_) from the dying cell into the tumor microenvironment [183,184]. This caspase-mediated tumor repopulation may also explain why caspase-3 expression sometimes correlates with cancer aggressiveness and poorer prognosis [185,186,187]. This “onco-regenerative niche” could potentially also be driven by the cargo present in apoptotic bodies or apoptotic cell-derived extracellular vesicles [188]. 

In the second instance, sublethal apoptotic signaling could provoke genomic instability via mutagenesis, potentially leading to the oncogenic transformation of non-cancerous cells and increasing the risk of de novo or subsequent cancer formation [45]. For example, irradiated or c-Myc over-expressing MCF10A cells formed tumors upon subcutaneous injection in nude mice only when caspase-3 was expressed [175,176]. ABT-737 induced cellular transformation, as indicated by anchorage-independent growth in soft agar, but this did not occur when caspases or MOMP were inhibited [168]. The mutational signature of subsequent “therapy-related” cancers often represents somatic mutations that originate from the damage to DNA generated by genotoxic therapies and the specific repair mechanisms that incorrectly fix these lesions [189]. Accordingly, sublethal CAD activation may also generate DSBs that could likewise create an oncogenic mutational signature. Similar to the *MLL* rearrangements underlining the de novo and therapy-induced myeloid leukemias discussed earlier, higher order chromatin fragmentation carried out by apoptotic nucleases could also generate *MLL* rearrangements [190]. Cleavage within the leukemogenic bcr of *MLL* and *AML1* identified following topoisomerase-II inhibition was also observed upon treatment with apoptotic stimuli that do not target topoisomerases, directly implicating fragmentation by apoptotic nucleases [191,192,193]. The fragmentation of DNA during apoptosis occurs non-randomly throughout the genome and sometimes localizes at topoisomerase cleavage sequences, such as *MLL*-bcr hotspots [194,195]. Indeed, anti-Fas treatment generated *MLL* translocations that were detected in daughter cells, suggesting that cells bearing FasL-induced *MLL* rearrangements survived and divided [196]. Inhibiting caspase activity suppressed *MLL*-bcr cleavage as well as the transcription of the *MLL-AF9* fusion gene upon treatment with anti-Fas [192,194,196]. Given the contribution of CAD to the mutagenesis upon death receptor ligation [161,162], the lack of *MLL* cleavage in caspase-deficient cells probably resulted from minimal ICAD cleavage and hence low-level CAD-generated DSBs. In support of this, *MLL* cleavage frequency was reduced or absent in CAD-deficient MEFs or cells over-expressing ICAD, directly implicating CAD in causing breaks within this gene [197,198]. DSBs generated by nucleases such as CAD activate error-prone NHEJ pathways that usually perform these rearrangements. H2AX was phosphorylated primarily by DNA-PK following TRAIL treatment [160]. Mutation frequencies were dramatically reduced in DNA-PKcs-deficient cells following exposure to chemotherapies doxorubicin and cisplatin, and vincristine, the mutagenesis of which was caspase/CAD-dependent [199]. This further highlights the mutagenic potential of NHEJ machinery and also directly implicates the requirement of error-prone repair pathways in CAD mutagenesis. As expected, DNA-PKcs was bound to the *MLL* cleavage site after irradiation and its inhibition increased the degree of DNA fragmentation, indicating that NHEJ can rapidly repair fragmented DNA [200]. Thus, the mis-repair of CAD-mediated DSBs by error-prone NHEJ probably underlines potential oncogenic genomic rearrangements following sublethal activation of apoptotic pathways. 

*MLL*-bcr cleavage was also attributed to EndoG in a caspase-independent setting when cells experienced enhanced replicative stress [201]. This supports an earlier report implicating EndoG and AIF (apoptosis-inducing factor, also released upon mitochondrial damage) in mediating *MLL* cleavage that did not involve caspase-3 and correlated with early, higher order chromatin condensation, a fragmentation step prior to CAD action [193]. Since hematopoietic stem cells (HPSCs) are more prone to replication fork stalling and DSB formation than terminally differentiated blood cells (especially following exposure to DNA-damaging agents) [202,203], activated EndoG in this context could encourage leukemic transformation via *MLL* rearrangements. This is important as proteins involved in NHEJ and single-strand annealing recombination (both error-prone) but not gene conversion HR (high fidelity) were frequently more active in HPSCs [203]. Interestingly, components for base excision repair (BER) were localized to regions of EndoG-mediated *MLL* cleavage after treatment with the DNA polymerase inhibitor aphidicolin, rather than NHEJ proteins [204]. This may be because EndoG can process both single- and double-stranded DNA or probably reflects the contribution from other *MLL* cleavage-causing factors upon replication stress, such as activation-induced cytidine deaminase during transcription, which can also localize to *MLL-*bcr active areas [204,205]. Furthermore, BER commonly repairs oxidative stress-induced DNA damage [206], and oxidative stress as a result of reactive oxygen species (ROS) can promote mitochondrial damage [207]. Given that EndoG normally resides in the mitochondria but localizes to the nucleus upon mitochondrial membrane permeabilization, the link between BER, NHEJ, and EndoG is not surprising. Indeed, inhibition of BER impacted on EndoG function [208]. 

Aside from the genesis of newly transformed clones via mutagenesis that affects tumor suppressor genes or oncogenes (spurring the formation of “treatment-induced” cancers), mutagenic sublethal apoptotic signaling may also contribute to intra-tumoral heterogeneity, possibly through the selection of pre-existing clones that harbor growth advantages: a “treatment-mediated” consequence of genotoxic therapies [78]. Genome sequencing suggested that treatments leading to t-AML may not necessarily result from direct *TP53* mutations but rather the selective pressure provoked by DNA-damaging agents that probably selected for pre-existing ‘chemo-resistant’ hematopoietic progenitor cells with sensitive genomes that further acquired leukemogenic changes after rounds of clonal expansion [209]. Exposure to chemotherapies may facilitate the rapid expansion of these clones that can persist long after treatment and may emerge as a subsequent cancer [210]. Chemotherapy- or apoptosis-induced fractional killing may provide the platform for the emergence of these apoptosis-resistant subpopulations [211,212]. It is not yet clear whether this selection phenomenon also contributes to the development of subsequent cancers derived from non-hemopoietic cell lineages that tend to be less severely impacted by anti-cancer therapy, which would presumably not be subject to such intense selective pressure. It is therefore difficult to confidently attribute proportions of the excess risk of different types of subsequent, independent cancers to the mutational activity of anti-cancer therapies versus their ability to impose selective pressure, although the mechanistic studies discussed earlier support a role for treatment-induced mutations. It is tempting to speculate that apoptotic signaling may also facilitate oncogenesis via this mechanism.

## 4. Mutagenic Consequences of Necroptotic Signaling

### 4.1. Necroptosis

Necroptotic cell death is a form of regulated necrotic death that stimulates immune activation. Depending on the status or expression of certain downstream proteins, TNFα-mediated activation of its receptors can stimulate pro-survival signaling pathways [213], apoptosis, or necroptosis [214], and these are largely mediated by receptor-interacting protein (RIP) kinases. Interferon receptors, Toll-like receptors (TLRs), or other RHIM-domain-containing proteins have also been described to mediate necroptotic death [215]. Furthermore, necroptosis may be initiated in virally or bacterially infected cells, or in cells subjected to physical or chemical trauma [216]. Necroptotic signaling following ligation of TNFR1 is well-characterized. Binding of TNFα promotes recruitment of the adaptor TRADD to the cytoplasmic death domain of TNFR1, enabling the formation of complex I containing RIPK1, TRAF2, and cIAP1/2 [217]. This complex can activate NFκB transcription to promote cell proliferation and survival via the interaction of NEMO with polyubiquitinated RIPK1 to activate the IκK complex. A non-canonical NFκB pathway can also emanate from the TNFR1 that is independent of RIPK1 [99]. Here, the cIAPs act as E3 ubiquitin ligases and are responsible for adding K63 polyubiquitin chains to RIPK1 (and other RIP relatives) [218]. In the absence of pro-survival signaling (such as when cIAP1/2 is degraded by Smac/DIABLO or drugs that mimic its function), RIPK1 is deubiquitinated and instead associates with FADD and caspase-8, transitioning from pro-survival complex I to pro-apoptotic complex IIa. This complex promotes apoptosis and caspase activation, although some cells can achieve this independently of RIPK1 [219,220]. In cells with low levels of caspase-8 or in the presence of a caspase inhibitor, RIPK1 associates with RIPK3 via their RHIM domains to form a pro-necroptotic complex IIb, which then mediates the phosphorylation of the mixed-lineage kinase domain-like (MLKL) pseudo-kinase [221]. Phosphorylated MLKL can then oligomerize and translocate to the plasma membrane to form membrane pores that release cellular contents, resulting in necroptotic demise of the cell [222,223]. Given that necroptosis is a caspase-independent form of cell death, it may represent an effective anti-tumor alternative to classical anti-cancer therapies that activate apoptotic machineries that may be defective in chemo-resistant cells [224], and may also avoid the mutagenic and possibly oncogenic effects of sublethal caspase signaling discussed earlier. 

### 4.2. Mutational Status of Cells Surviving Necroptotic Signaling

Much research is currently being carried out to fully understand all aspects of necroptosis, so relatively little is presently known about the direct mutagenic consequences of necroptotic signaling. Since cells can survive apoptotic signaling, are there also mechanisms in place that allow cells to survive necroptotic signaling? MLKL-mediated membrane rupture was initially considered a “point of no return” in cell necroptotic fate. However, just like the previous notion that caspase activation was considered a lethal event (which we now accept is not the case as cells can withstand sublethal levels of active caspases), emerging evidence challenges this ‘always-fatal’ function of MLKL. Gong et al. [225] found that induction of necroptosis via the expression of a dimerizable active RIPK3 or MLKL mutant, or upon TNF/zVAD-fmk treatment, activated MLKL and induced phosphatidylserine (PS) exposure on the cell membrane prior to membrane disruption. A similar observation was made in wild-type fibroblasts treated with IFNγ/zVAD-fmk or caspase-8-deficient fibroblasts treated with IFNγ [226]. Gong et al. [225] describe the “resuscitation” of PS-exposed, membrane intact cells experiencing activated and membrane-localized MLKL by the function of ESCRT-III components. In this context, the ESCRT machinery most likely engages membrane repair as ESCRT components localized at MLKL damage sites on the plasma membrane, and silencing of ESCRT genes sensitized cells to necroptosis [227,228]. This delayed or even prevented MLKL-mediated loss of plasma membrane integrity, enabling cell survival following necroptosis [225]. Indeed, PS-exposed, membrane intact cells that were sorted and cultured in media without the initial necroptotic stimulus regained PS asymmetry, inactivated MLKL, and remained viable [225,226]. Furthermore, intact and PS-exposed necroptotic cells sorted and grown in media containing necrosulfonamide to inhibit active phosphorylated MLKL [229] survived longer post-sorting than cells without necrosulfonamide treatment, illustrating delayed death following MLKL activation [230]. Another way in which a cell could conceivably withstand active MLKL may be through its sequestration and release in necroptotic bodies. Extracellular vesicle bodies shed during necroptosis consisted of cargo that was rich in phosphorylated MLKL, and this reduced the levels of cellular phosphorylated MLKL, delaying the onset of necrosis [228,230]. These studies illustrate that cells have mechanisms to slow down or reverse the cytotoxicity of activated MLKL. A proposed physiological role for delayed necroptotic death may be to maximize immune stimulation by allowing extra time for immunogenic signals to attract immune cells, for example with longer PS exposure or the release of pro-inflammatory cytokines, prior to complete demise of the cell [225]. 

Given the evidence describing cell “resuscitation” following necroptosis, are there mutagenic consequences to sublethal necroptotic signaling? Recent in vitro analysis determined that classic activation of necroptosis via TNFα, caspase-8 inhibition, and IAP antagonism failed to provoke DNA damage or mutations in surviving cells [231]. Importantly, DNA damage was not detected in cells expressing a lethal constitutively active MLKL mutant, implying that the execution of necroptosis by MLKL was not genotoxic. 

While DNA damage does not appear to be associated with necroptotic death, p53-independent ripoptosome assembly and subsequent apoptosis or necroptosis can occur in response to DNA-damaging stimuli in some cell types [232,233]. Etoposide-induced ripoptosome formation initiated caspase-8-mediated apoptotic or RIPK3-mediated necroptotic cell death [232,234]. Mechanistically, RIPK1 is critical for propagating this cell death signal, and the formation of these pro-death signaling complexes are likely facilitated by the decreased expression of cIAPs and cFLIPs that accompanies genotoxic stress [235,236,237]. ATM but not p53 was required for NEMO association with RIPK1 in cells exposed to high doses of etoposide, which led to the recruitment of FADD and caspase-8 [234,238,239]. Cytoplasmic retinoic acid receptor-γ was recently reported to associate with RIPK1 following cisplatin or etoposide treatment, and this allowed for ripoptosome-mediated cell death [233]. DNA damaged-induced autocrine TNFα production via RIPK1-mediated NFκB activation also contributed to cell death in these contexts although this differed depending on stimulus and cell type [240,241]. For instance, 5-FU-treated colon cancer cells underwent necroptosis upon caspase inhibition that was mediated by autocrine secretion of TNFα [242], while autocrine TNFα was only partially required for cisplatin-induced lethality in L929 cells [241]. Instead, cisplatin could mediate TNFα-independent necroptosis following mitochondrial permeability transition pore formation and ROS generation upon the RIPK1/RIPK3/MLKL necrosome. The significance of this goes back to earlier discussions: genotoxic stimuli can provoke mutations either via direct effects on DNA or indirectly via sublethal apoptotic activation of CAD. Theoretically, DNA-damaging stimuli could still directly provoke mutations in cells that activate necroptotic signaling if necroptotic effectors were blocked or if cells withstood modest levels of MLKL activation. The mutagenesis that may then arise from sublethal necroptotic signaling hypothesized here would be different from CAD-mediated mutagenesis associated with sublethal apoptotic signaling, as the initial DNA damage would be attributed to the genotoxic stimulus rather than an indirect effect of the necroptotic pathway. The fact that cellular “resuscitation” from MLKL activation has been described suggests that genotoxicity-induced necroptosis could result in genomic instability in necroptosis-surviving cells. It would be interesting to discern any differences in mutation frequencies upon DNA-damaging stimuli that induce apoptosis versus necroptosis, especially given that some mutations following topoisomerase inhibition, for example, were CAD-dependent [162].

**Figure 2:**
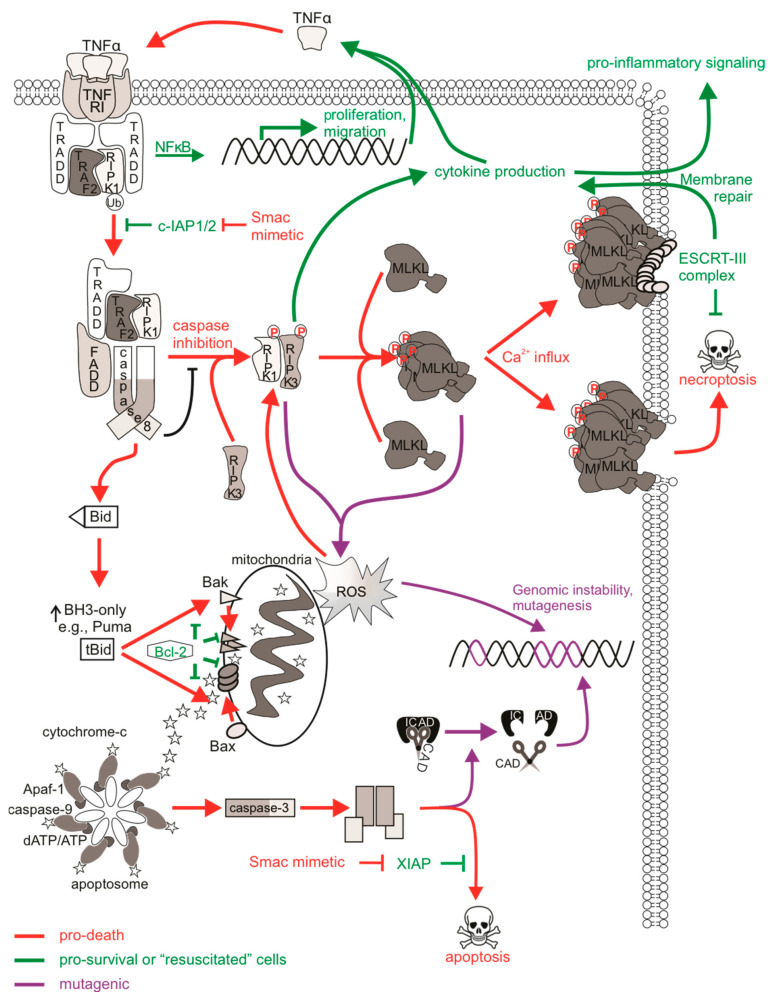
The mutagenic potential of necroptotic signaling. cIAP1/2 acting at TNFR1 complex I polyubiquitinates RIPK1 to allow for NFκB transcription and expression of proliferative and migratory genes. Some cells can autocrine produce TNFα to further stimulate TNFR1 signaling. There is a transition to pro-death complexes IIa (if caspase-8 is present) or IIb (if caspase-8 activity is inhibited) upon incapacitation of cIAP1/2. Pro-apoptotic ripoptosome formation leads to MOMP and caspase activation, which can provoke mutations via CAD-dependent DNA damage. The pro-necroptotic necrosome is formed in cells lacking caspase-8 activity allowing RIPK1 recruitment of RIPK3, the autophosphorylation of RIPK3, and the subsequent phosphorylation and activation of MLKL by RIPK3. Activated MLKL oligomerizes then translocates to the plasma membrane, where it forms pores to compromise membrane integrity. The ensuing MLKL-mediated necroptotic death is not associated with DNA damage. The initiation of ESCRT-III-mediated membrane repair of MLKL pores delays the onset of or even prevents necroptotic cell lysis and allows for RIPK3-dependent cytokine production and release. Survival following necroptotic signaling is known as “resuscitation”. The necrosome can also stimulate mitochondrial-dependent or independent ROS production, which can stabilize the RIPK1/RIPK3 necrosome as a positive feedback loop. DNA can be directly impacted by ROS, and mutagenesis might occur if damage is mis-repaired or there is sustained genomic instability in “resuscitated” cells.

While survival from non-genotoxic necroptotic stimuli may be a promising non-mutagenic feature of necroptosis, the activation of upstream necroptotic components upon necroptotic signaling may have unwanted consequences that may contribute to cancer initiation and progression or other disease [243]. Pro-inflammatory cytokines such as TNFα, CXCL2, CXCL8 and CXCL11 are released upon activation of necroptotic proteins, probably via Erk/MAPK and NFκB transcriptional pathways [244,245]. RIPK1 can function to stimulate TNFα production by NFκB-dependent and independent pathways [213], and the secretion of chemokines and other immunoregulatory molecules occur in a RIPK3- and MLKL-dependent manner during necroptosis [225,246]. RIPK3 was also reported to aid in formation of the inflammasome, leading to caspase-1 mediated release of IL-1β [247], concurrent with the immune-stimulatory effects of necroptosis. Interestingly, cytokine induction reportedly occurred in a cell-autonomous manner rather than indirectly from the release of damage/danger-associated molecular patterns (DAMPs) [244,246], meaning that this regulated release of key cytokines occurs prior to necroptotic cell lysis. This is critical as it implies that sublethal activation of RIPK1, RIPK3 or MLKL could still achieve cytokine induction and immune activation, which could manifest in “resuscitated” cells. In addition, necroptosis can also trigger an adaptive immune response. For example, dendritic cells engulf necroptotic corpses and trigger the activation of CD8+ T cells via cross presentation upon PS exposure and chemoattraction [246,248,249]. However, these responses usually occur in dying cells upon the release of DAMPs but require RIPK1 and NFκB signaling [250]. The oncogenic consequence here (upon a necroptotic or “resuscitated” cell) would be the potential for excess inflammatory stimulation of surrounding cells, leading to a possible pro-inflammatory, pro-tumorigenic environment [251]. TNFα is known to lead to such pathologies by activating signaling pathways that promote survival, proliferation, and invasion [252]. Cytokine release syndrome is a dose-limiting side effect of Smac mimetic treatment in patients due to the over-production of TNFα upon cIAP degradation and subsequent ripoptosome formation [253]. This type of “therapy-induced inflammation” may represent an inflammatory side effect of surviving cells experiencing necroptosis-mediated cytokine induction in addition to tumor neo-antigens or DAMPs released from dying tumor cells [254]. Further to this, if TNFα-mediated signaling were to occur in surrounding cells and extrinsic apoptotic pathways were activated to sublethal levels, then this may provoke mutagenesis via CAD [231] or oxidative stress from ROS [255] (Figure 2).

Redox regulatory roles of necroptosis have been reported, and thus the oxidative stress associated with these processes may have mutagenic consequences. Necrosome formation enhanced mitochondrial ROS (mtROS) accumulation upon TNFα-mediated cell death [256,257]. RIPK3 and RIPK1 appear to contribute more than MLKL to mtROS production [258], probably as a result of kinase function. For example, RIPK3 can phosphorylate the mitochondrial pyruvate dehydrogenase complex to enhance aerobic respiration, leading to mtROS, which acts as a positive feedback loop to further encourage RIPK3 activity [259]. While this may be an important physiological response in pathogen-infected cells, for instance by promoting inflammasome activation [247,260], ROS modulation by necroptosis may contribute pathologies related to oxidative stress [261]. In addition, high levels of ROS can damage DNA via direct reactivity to the sugar backbone of DNA, thereby oxidizing nucleoside bases or modulating replication stress [262]. Necroptosis-mediated ROS production may then represent an indirect activation of DDR pathways associated with necroptosis. The resulting genomic instability could then persist in “resuscitated” cells experiencing these various forms of ROS-mediated DNA damage.

## 5. Mutagenic Consequences of Other Cell Death Signaling Pathways

### 5.1. Possible Mutagenic Consequences of Pyroptotic Signaling

Pyroptosis is a pro-inflammatory mode of cell death which acts as a defense mechanism against infection. It is the culmination of molecular pathways that respond to the activation of nucleotide-binding oligomerization (NOD)- like receptors (NLRs), which are pattern recognition receptors (PRRs) that sense internal danger signals, such as pathogen-associated molecular patterns (PAMPs) and DAMPs. TLRs, C-type lectins, and galectins fall under the NLR family of PRRs and participate in molecular complexes termed “inflammasomes” to stimulate immune activity and pyroptotic cell death [263]. Inflammasomes activate inflammatory caspases, promoting caspase-mediated maturation of cytokines IL-1β and IL-18. Upon recognition, sensor proteins (such as NLRP3) recruit the adaptor protein ASC, which aggregates to create a caspase-1 activation platform via proximity-induced auto-processing [264]. Active caspase-1 cleaves pro-IL-1β into its mature form as well as cytosolic gasdermin D (GSDMD), enabling the oligomerization of its N-terminal fragment, which forms ring-like pores on the plasma membrane to allow cytokine release and facilitate pyroptotic cell lysis [265]. Caspases-4 and -5 (or -11 in mouse) can directly bind LPS to stimulate non-canonical NLRP3 inflammasome activation [266,267]. This initiates caspase-1-mediated cytokine maturation and activation of GSDMD to induce pyroptosis. Caspase-8 also contributes to canonical and non-canonical NLRP3 inflammasomes in the absence of caspase-1 [268]. 

There are no reports to date defining any direct mutagenic effect of sublethal pyroptotic signaling, however emerging evidence describes the ability of cells to withstand sublethal levels of pyroptotic pathway activation. Pyroptosis is defined by the activation of inflammatory caspase-1 upon inflammasome formation, leading to the controlled release of IL-1 cytokines and lytic cell death. GSDMD-generated pores were initially believed to be sufficient for pyroptosis and concurrent IL-1β release, however the release of IL-1 cytokines from inflammasome-active viable cells, also known as “hyperactivated” cells, can also occur in the absence of pyroptosis, suggesting that mechanisms exist to regulate cell fate after inflammasome activation [269,270,271,272]. This is often depicted through the detection of IL-1β but not LDH (which is released from lysed cells) in culture media alongside the uptake of propidium iodide by means of GSDMD membrane pores. These “hyperactivated” viable cells containing active inflammasomes could be considered to manifest “sublethal” pyroptosis. Various situations have been reported in which cells exhibit this phenotype, including macrophages infected with *S. aureus* [273,274] and dendritic cells stimulated with oxidized phospholipids derived from dead cells [270,272]. The TLR SARM was recently reported to modulate pyroptosis-associated mitochondrial depolarization, leading to NLRP3 association with ASC and inflammasome activation [271]. In this context, cells experiencing greater mitochondrial depolarization displayed more NLRP3-caspase-1 inflammasome activation, leading to some IL-1β release and more pyroptosis, whereas cells lacking mitochondrial depolarization exhibited minimal NLRP3-dependent caspase-1 activation and were not pyroptotic, but still released IL-1β through GSDMD pores. This scenario—viable cells bearing active inflammasomes—could be considered “sublethal pyroptotic signaling”. These studies highlight the ability of cells to withstand inflammasome activation and inflammatory caspase activity, and some downstream processes, without succumbing to cell death.

The fact that GSDMD membrane pores can form in viable cells to allow cytokine release suggests that a threshold amount of activated GSDMD needs to be achieved in order to execute pyroptosis. Similar to the restoration of plasma membrane integrity in MLKL activated “resuscitated” necroptotic cells discussed earlier, ESCRT-mediated membrane repair can also follow GSDMD activation and pore formation, resulting in the delay or blockage of pyroptosis, and enhancing cell survival [275]. Indeed, the recruitment of ESCRT-III machinery to sites of GSDMD pores occurred in a calcium-dependent manner, which is a conserved mechanism of plasma membrane repair at sites of membrane damage [276]. It is postulated that the ability of ESCRT-III components to maintain membrane integrity by removing GSDMD pores and preventing pyroptotic lysis may enhance immune stimulation and pathogen clearance in some contexts, particularly given that cytokine release from “hyperactive” living cells triggers a stronger adaptive immune response [277].

DNA damage has been detected in cells bearing inflammasome activity [278,279,280]. However, whether this leads to mutations in surviving cells, or merely reflects DNA degradation in dying cells, has not been determined. The mechanism responsible for the DNA damage is also unclear. Intact ICAD was detected in treated and untreated cells [278,279], prompting researchers to argue that CAD was not activated via the inflammasome. However, caspase-1 can cleave ICAD (although relatively inefficiently) [281], so perhaps it is premature to exclude this mechanism.

**Figure 3:**
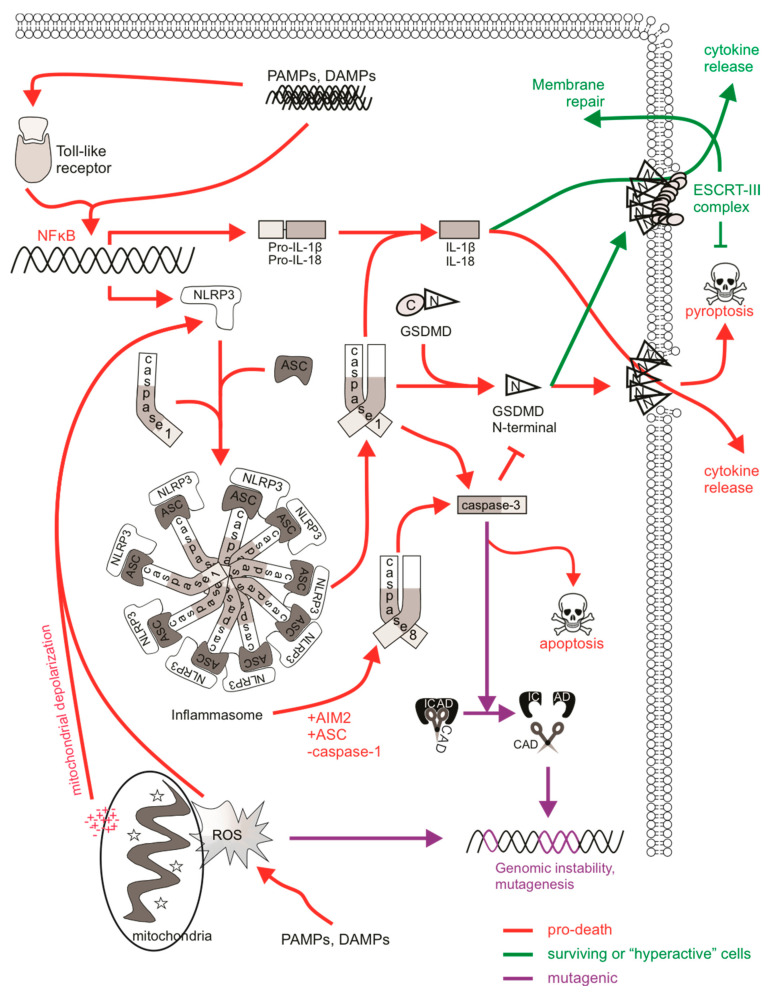
The mutagenic potential of pyroptotic signaling. PAMPs/DAMPs can directly or indirectly stimulate inflammasome formation via NFκB-mediated transcription. Sensing (e.g., NLRP3 or AIM2) and adaptor (e.g., ASC) proteins interact with caspase-1 to stimulate active caspase-1 function, which cleaves pro-IL-1β and IL-18 to their mature forms. Caspase-1 also cleaves gasdermin D (GSDMD), allowing its N-terminal fragment to translocate to the plasma membrane, where it oligomerizes and forms membrane pores. Mature cytokines and other cellular contents are released via these pores, wherein extensive osmotic influx leads to pyroptotic cell lysis. Some cells can be “hyperactive”, where they experience inflammasome formation, caspase-1 activation, cytokine maturation, and GSDMD cleavage, however cytokines are released without the cell succumbing to pyroptosis. This may be achieved via ESCRT-mediated plasma membrane repair. ESCRT-III machinery can repair GSDMD pores to delay the onset of pyroptotic cell lysis. Caspase-3 may also be cleaved and activated by caspase-1 or caspase-8 (in cells deficient in caspase-1), leading to apoptosis rather than pyroptosis. Caspase/CAD-mediated mutagenesis may occur in cells achieving sublethal levels of caspase-3. ROS, often derived from the mitochondria upon depolarization, can also stimulate NLRP3 inflammasome activation and pyroptosis. DNA can be directly impacted by ROS, and mutagenesis might occur if damage is mis-repaired or there is sustained genomic instability in “hyperactive” cells.

Apoptotic caspases can also be activated upon inflammasome formation. LPS stimulation of monocytes induced the formation of an NLRP3 “alternative inflammasome” complex, yielding active caspase-1, IL-1β release but no pyroptosis, and requiring TRIF-mediated RIPK1-FADD-caspase-8 ripoptosome formation [274]. Caspase-8 proteolytic activation and catalytic activity was detected upstream of NLRP3 activation, suggesting that caspase-3 processing by caspase-8 could occur, although caspase-3 mediated apoptotic signaling was not detected [274]. Cytosolic DNA activated the AIM2 inflammasome complex, which recruited caspase-8, leading to caspase-3-mediated apoptosis, or caspase-1 leading to pyroptosis [282]. Activated caspase-1 via the AIM2 inflammasome reportedly also cleaved caspase-3 [283], consistent with earlier reports that recombinant caspase-1 could cleave caspase-3 in vitro [284,285]. These suggest that the inflammasomes can activate DNA repair mechanisms to repair ROS-generated or possibly even CAD- or EndoG-mediated DNA damage in a caspase-3-dependent or independent manner. It is therefore possible for caspase-1-mediated sublethal activation of caspase-3 to promote genomic instability in inflammasome active, non-pyroptotic cells if NLRP3-mediated activation of DNA repair pathways initiates mis-repair in error-prone cells (such as those with defective high-fidelity repair) surviving pyroptotic signaling (Figure 3). 

The stimulation of inflammasome formation upon mitochondrial depolarization indicates that redox regulation of NLRP3 can occur, probably via the release of mtROS [286,287,288]. Oxidative stress from mtROS can impact genomic integrity, implying a possible link between pyroptotic signaling and DNA damage responses. The ability of EndoG to fragment mitochondrial DNA (mtDNA) in this context suggests that it could also process genomic DNA if localized to the nucleus, particularly upon mitochondrial damage [208]. Monosodium urate (MSU) crystals increased mtROS, stimulated NLRP3 inflammasome activation, provoked DNA damage and enhanced the expression of double-strand and base excision DNA repair and apoptotic genes in wild-type dendritic cells. However, their expression was lower in NLRP3- or caspase-1-deficient cells suggesting that key inflammasome components permit DDRs [289]. DAMP-stimulated macrophages activated NADPH oxidase and TLR4/MyD88-dependent signaling, triggering mtROS formation. Interestingly, EndoG was also activated and fragmented mtDNA [290]. An association between NLRP3 and ATM has been reported: DNA damage triggered p53-mediated intrinsic apoptosis via the direct binding of NLRP3 to ATM, allowing for its activation, while ATM-dependent DNA repair signaling was reduced in NLRP3-depleted cells [291]. Non-canonical DDR and ATM activation have been described upon viral infection, among other stimuli, suggesting that the oxidative stress in infected cells can induce both inflammasome activation and DNA damage [292,293]. Therefore, it could be possible for oxidative stress-induced DNA damage to be sustained in inflammasome-active, non-pyroptotic cells. 

### 5.2. The Role of DDR in Ferroptosis

Ferroptosis is a unique form of non-apoptotic, oxidative regulated cell death driven by iron-dependent lipid peroxidation and increased redox imbalance [294]. Extensive lipid peroxidation in ferroptosis can occur upon inhibition of glutathione peroxidase (GPX4), which requires the cofactors glutathione (GSH) and NADPH, and as such, depletion of GSH or NADPH impedes GPX4’s function to induce ferroptosis [295]. Additionally, inhibition of system x_c_^−^, particularly the xCT transmembrane anti-transporter which mediates extracellular cystine import in exchange for intracellular glutamate, can also trigger ferroptosis as cystine is a precursor for GSH synthesis [296]. Ferroptosis has been associated with various diseases, particularly those highlighted by excessive oxidative stress or redox imbalances such as neurodegenerative diseases and cancer [297].

Even though ferroptotic stimuli do not appear to directly induce DNA damage (as they target cellular components that modulate cysteine, GSH, or lipid levels), key proteins involved in the DDR are involved in the regulation of ferroptosis, although the role of p53, for example, in ferroptosis is highly context-dependent [298]. A metabolic function of p53 in transcriptionally regulating cystine uptake or lipid metabolism was implicated in ferroptosis, separate from p53’s roles in DNA repair and apoptosis [299,300]. Silencing or pharmacological inhibition of ATM (or ATR) offered protection from ferroptosis through regulating expression of iron regulators ferritin, ferroportin and MTF1 [301]. Even though p53 is an important downstream target of ATM and has been implicated in regulating ferroptosis, Chen et al. [301] did not observe a contribution of p53 (or Chk2) to ferroptosis, in contrast to ATM inhibition, implying a non-canonical function of ATM. Irradiation was reported to sensitize cells to ferroptosis, probably due to lipid peroxidation induced here [302] or via the ability of ATM to function in response to the oxidative stress upon radiation [303], possibly linking genotoxic stimuli to ferroptosis. These studies illustrate the non-canonical activation of DDR proteins in the absence of classic DNA damage during ferroptosis [304].

The accumulation of lipid ROS and mtROS resulting from the dysregulation of mitochondrial metabolism caused by ferroptotic stimuli indicates a strong link between ferroptosis and oxidative stress [305,306]. As indicated earlier, oxidative stress as a result of large quantities of ROS can promote DNA damage and is sometimes lethal. Stabilized p53 delayed the onset of ferroptosis in response to cystine deprivation by transcriptionally upregulating p21^Waf1/Cip1^ to inhibit cell-cycle progression in order to conserve intracellular glutathione and reduce lipid ROS accumulation [307]. Furthermore, p21^Waf1/Cip1^ expression and activity in cancer cells correlated with sensitivity to ferroptosis, although this effect may be independent of p53 [308]. This may represent a context in which cell cycle arrest enabled a cell to withstand ferroptotic conditions to allow replenishment of key proteins critical for surviving ferroptosis. It may therefore be possible for some generated ROS sustained during such a period of conservation to exert mutagenic effects on DNA.

## 6. Concluding Remarks

**Figure 4:**
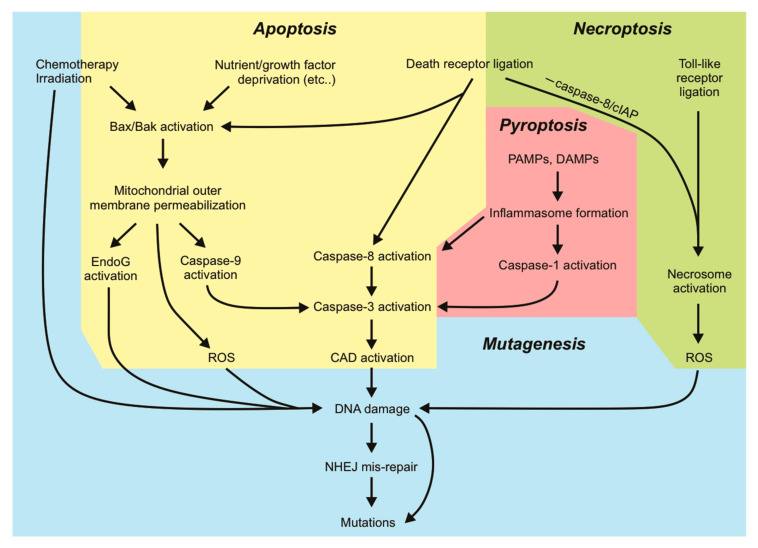
Summary of the key aspects of cell death pathways that can lead to mutagenesis.

Improvements in outcomes for some cancer types have prompted clinicians and researchers to focus on the more nuanced goal of sparing cured patients severe acute and late adverse effects of therapy. A well-recognized late effect of anti-cancer treatment is subsequent cancers, which can arise, at least in part, due to the mutational activity of traditional anti-cancer therapies. Ionizing radiation and certain chemotherapeutic drug classes, such as topoisomerase-II poisons, induce chromosomal changes that enhance a patient’s risk of subsequent oncogenesis. It is now possible to trigger cancer cell death directly, by engaging cell death pathways, rather than indirectly by creating DNA damage which secondarily stimulates cancerous cells to die. This prospect raised the possibility that therapy-induced cancers could be avoided. Unfortunately, this hope has been tampered, at least as far as direct apoptosis-inducing drugs are concerned, by the realization that pro-apoptotic stimuli can promote genomic instability via caspase-mediated activation of nucleases (Figure 4). Activation of extrinsic apoptotic signaling by TRAIL receptor agonists provoked mutations in surviving cells [161,162], and the minority MOMP and subsequent caspase-mediated mutagenesis provoked by BH3-mimetic drugs facilitated oncogenic transformation in vivo [168]. TRAIL receptor agonists have not yet been approved for clinical use [309], and the clinical use of such BH3 mimetics are still in relatively early stages, so the frequency of subsequent cancers in patients treated with these agents will not be evident for many years.

Drugs that promote death by necroptosis may avoid many of the pro-survival advantages that apoptosis-resistant cancer cells rely on [214]. Due to this, these drugs may also be less likely to exert the same selective pressure as chemotherapy drugs, which were described to boost the frequency of pre-existing clones harboring pro-cancerous mutations in tumor suppressor genes such as *TP53*. The observation that sublethal necroptotic signaling was non-mutagenic provides hope that drugs which destroy cancer cells via necroptosis may be less prone to trigger development of second cancers than chemotherapy or radiotherapy, or possibly even than direct apoptosis inducers. This feature may be especially beneficial for cancer patients with germline flaws in DNA damage response pathways, for whom mutagenic therapies would be particularly risky. As yet, no anti-cancer agents have been created that exclusively trigger necroptosis, but Smac mimetic treatment (which can provoke apoptotic or necroptotic cell death) was non-mutagenic in vitro, even when accurate DNA repair was compromised [199]. Further research will be needed to define the mutagenicity associated with sublethal activation of more recently described cell death pathways, including pyroptosis and ferroptosis, and the prospects for therapeutic exploitation of these pathways.
